# Association of the Risk of Osteoarthritis and Hypertension in the Korean Adult Population Aged 40–59 in Pre- and Postmenopausal Women: Using Korea National Health and Nutrition Examination Survey 2012–2016 Data

**DOI:** 10.1155/2021/8065838

**Published:** 2021-02-23

**Authors:** Mikyung Ryu, Ji Sun Ha, Sol Lee, Weon-Chil Baek, Heejin Kimm, Ho Gym

**Affiliations:** ^1^Department of Sports and Health Science, College of Human-Centered Convergence, Kyonggi University, Suwon, Republic of Korea; ^2^Institute on Aging, Ajou University Medical Center, Suwon, Republic of Korea; ^3^College of Nursing Science, Kyung Hee University, Seoul, Republic of Korea; ^4^Department of Bbko Research Center, Bbko Big-Data R&D, Seoul, Republic of Korea; ^5^Department of Health Policy and Management, Korea University, Seoul, Republic of Korea; ^6^Department of Epidemiology and Health Promotion, Institute for Health Promotion, Graduate School of Public Health, Yonsei University, Seoul, Republic of Korea; ^7^Department of Epidemiology and Health Promotion, Graduate School of Public Health, Yonsei University, Seoul, Republic of Korea

## Abstract

**Purpose:**

Previous studies reported the relation of osteoarthritis (OA) and hypertension (HTN) mostly in postmenopausal women. This study aimed to identify the association between OA and HTN in pre- and postmenopausal women.

**Methods:**

We used data of 4,627 middle-aged (40–59 years) women from the Korea National Health and Nutrition Examination Survey (KNHANES) from 2012 to 2016. Chi-square and *t*-test compared the characteristics of the participants. Binomial logistic regression was used to identify an association between OA and HTN under controlling covariates such as age, tobacco smoking, alcohol consumption, and obesity.

**Results:**

There were 1,859 participants with non-OA and menopause, 104 with OA and nonmenopause, and 375 with OA and menopause, respectively. The number of women with OA and HTN was 129. OA was significantly associated with HTN diagnosis in postmenopausal women under controlling covariates (odds ratio: 1.408, 95% CI: 1.092–1.815, *p*=0.008). However, this relationship was weakened in premenopausal women (odds ratio: 1.651, 95% CI: 0.950–2.869, *p*=0.075).

**Conclusion:**

In conclusion, women with HTN showed a distinct association with OA than the normotensives, and this relationship was more apparent among postmenopausal women. Further research is needed for a preventive approach.

## 1. Introduction

At middle age in the life cycle, chronic diseases begin, but preventive intervention is still applicable. There is a qualitative difference in middle-aged women's health, depending on whether they are menopause (Mp) because hormone metabolism changes. As the circulating estrogen decreases, the risk of HTN and cardiovascular disease increases [1], and gender difference became remarkable in terms of the deteriorated degenerative changes in the articular cartilage [2]. Therefore, menopausal status in the two major chronic diseases, HTN and osteoarthritis (OA), makes a fundamental difference.

OA, a highly prevalent disease in the elderly and severe joint disease at the same time because of deterioration and inflammation of the articular cartilage, has been known as the major leading cause of disability with activity restriction and pain resulting in low quality of life as well as a higher rate of hospitalization [3, 4]. Moreover, the aging population has been reported to be exposed to painful disabling OA leading to higher therapeutic costs [4–6]. In several previous pieces of research, tobacco smoking, divorce, unemployment [7], old age, sex, obesity, low educational level, low strength exercise frequency, manual labor, and HTN [8, 9] were reported as potential risk factors for OA [6–9].

HTN has been reported as a primary and most common independent risk factor for cardiovascular disease, as well as one of the metabolic syndrome components, and then as the 3rd major cause of disability worldwide [10, 11].

Association between high blood pressure and OA has been reported [8, 9], but few studies have been conducted on this topic. In a cross-sectional study of men and women over 50 years old, the risk of OA in patients with hypertension was 1.394 times (95% CI: 1.052–1.846) [8]. In a community survey for over 50, the prevalence of OA in women was three times that of men, and hypertension was associated with knee osteoarthritis [9]. Hart et al. suggested a significant association between OA and metabolic syndrome, including hyperglycemia and hyperlipidemia, based on an analysis with women aged 45–64 [12]. This result supports the hypothesis that OA can be described as a metabolic factor in its mechanism, as suggested by Velasques and Katz [13].

However, most of the above studies seem to include only postmenopausal women. On the contrary, Inoue et al. [14] studied community participants aged 30–86 years, and the prevalence of hypertension in the OA group was higher in both men and women, but the relationship with the metabolic syndrome was found only in women [14]. These results suggest that the relationship between OA and HTN was also raised in premenopausal women. However, we could not find any analysis considering menopause in the above studies.

Also, it has been challenging to find a study that explores the relationship between hypertension and OA, considering menopause, apart from metabolic syndrome in middle-aged women rather than the elderly. In middle age, the number of women diagnosed with hypertension increases. Therefore, if there is a link to the risk of OA, then this is an appropriate time to implement preventive behavior for joint protection. Besides, in clinical practice, information about risks and preventive skills according to each woman's life cycle is needed. So, it is necessary to find out the difference due to the menopausal status.

Therefore, this study aims to identify the association between OA and HTN with the population of middle age, 40–59 years, in pre- and postmenopausal women using the Korea National Health and Nutrition Examination Survey (KNHANES) data.

## 2. Methods

### 2.1. Study Population

This cross-sectional study used the Korea National Health and Nutrition Examination Survey (KNHANES) from 2012 to 2016, performed by the Korea Centers for Disease Control and Prevention (KCDC). Kweon et al. described the details of the survey design and data resource profile in 2014 [15]. KNHANES is nationwide representative data using two-step stratified clustered equal-probability sampling. As a first step, the population was stratified into 16 administrative areas, and in the second step, it was stratified into 26 substrata according to age and sex in general residential areas. Investigation clusters were selected from the strata, and about 20 households were selected in each cluster. All family members of the households who were older than one year were invited into the survey [15, 16]. The institutional review board approved the protocols of this study of the KCDC with obtaining written informed consent forms from all participants. KNHANES data from 2012 to 2016 included the survey on health interviews, health examination, and nutritional assessment. We selected female participants more than 40 years old and less than 60 years old who completed the evaluations, such as HTN diagnosis (yes or no), osteoarthritis diagnosis (yes or no), and menopause (yes or no). A total of 4,627 female participants were finally involved in the analysis.

### 2.2. Identification of Osteoarthritis and Menopause Status

It was confirmed about OA diagnosis if participants have been diagnosed with OA by physicians or not in health survey questionnaires in KNHANES. We excluded the patients with no answer or nonapplicable in the questionnaire. Menopause included both natural and artificial menopause. Artificial menopause means menopause by bilateral ovarian removal, and natural menopause means that normal menstruation stopped for more than one year due to a decline in ovarian function.

### 2.3. Characteristics of the Participants

The following baseline sociodemographic characteristics were assessed: age with ≥40 years and <60 years, smoking status classified as none, past, or current, alcohol consumption classified as nondrinker + under one glass per month or over one glass per month, menopause classified as yes or no, HTN diagnosis classified as yes or no, and BMI classified as low (<18.5 Kg/m^2^), normal (18.5 to <25.0 Kg/m^2^), or obesity (25.0 or higher Kg/m^2^). Korean version of the euro quality of life-5 dimensions (EQ-5D) is a composite measure of health outcomes consisting of mobility, self-care, daily activity, pain/discomfort, and anxiety/depression with each factor containing three-level health status [17]. In laboratory data, systolic blood pressure (SBP, mmHg), diastolic blood pressure (DBP, mmHg), fasting blood sugar (FBS, mg/dL), triglyceride (TG, mg/dL), cholesterol (Chol, mg/dL), high-density lipoprotein cholesterol (HDL-C, mg/dL), aspartate aminotransferase (AST, IU/L), alanine aminotransferase (ALT, IU/L), and creatinine (CREA, mg/dL) were involved.

### 2.4. Statistical Analyses

The data were expressed by numbers and percentages for describing the general characteristics of the study participants, and the frequency in each group (OA or non-OA, Mp or PreMp, and HTN or non-HTN) was compared by the chi-square test. The proportion was estimated using weights and “survey” procedures according to the analysis protocol provided by the Korean CDC, which accounted for the proportion of selection and proportion of response [16]. An independent *t*-test was performed to determine the difference between clinical variables in each group. Binomial logistic regression was used to identify an association between OA and HTN with the participants aged 40–59, in total, PreMp and Mp women by determining odds ratios (ORs) and 95% confidence intervals (CIs), under controlling covariates such as age, tobacco smoking, alcohol consumption, and obesity as categorical variables. The significance level was less than 0.05 for *p* values. We used IBM SPSS Statistics 22.0 software (IBM Corp, Armonk, NY).

## 3. Results

### 3.1. Characteristics of the Study Population

A total of 4,627 female participants were included in this study. Overall mean age was 49.7 (5.8) years old, 49.2 (5.7) in 4148 non-OA participants and 53.9 (4.3) in 479 OA participants. There were 2,289 (55.2%) participants with non-OA and nonmenopause, 1859 (44.8%) with non-OA and menopause, 104 (21.7%) with OA and nonmenopause, and 375 (78.3%) with OA and menopause, respectively. In HTN diagnosis, 3,604 (86.9%) participants were with non-OA and non-HTN, 544 (13.1%) with non-OA and HTN, 350 (73.1%) with OA and non-HTN, and 129 (26.9%) with OA and HTN, respectively. Other results are shown in Table 1.

### 3.2. Characteristics of the Premenopause and Menopause Groups according to the Osteoarthritis Status

In both non-OA and OA groups, the Mp group had a significantly higher age, more HTN, and less alcohol consumption than the PreMp group. In the non-OA group, there were significantly more nonsmokers in the Mp group (*p*=0.042), and there were also more obese subjects (*p*=0.004), but these were not significantly related with Mp in the OA group.

In both non-OA and OA groups, the Mp group had significantly higher SBP and higher cholesterol than the PreMp group. Mp subjects of the non-OA group showed higher DBP, FBS, TG, AST, and ALT, but they had lower HDL-C than the PreMp group. However, there was no significant difference in these clinical laboratory variables between PreMp and Mp subjects in the OA group. In both non-OA and OA groups, creatinine was not associated with the Mp status (Table 2).

### 3.3. Characteristics of the Groups with and without Hypertension according to the Menopause Status

In both PreMp group and Mp group, the HTN group showed significantly higher age, more OA, and more obesity cases. Smoking and alcohol consumption status were not associated with HTN in both groups.

In both PreMp group and Mp group, HTN patients had significantly higher SBP (*p* < 0.001), DBP (*p* < 0.001), FBS (*p* < 0.001), TG (*p* < 0.001), AST, ALT, and lower HDL-C (*p* < 0.001). HTN patients of the Mp group showed lower cholesterol than nonhypertensive subjects, but there was no significant difference in cholesterol according to HTN in the PreMp group (Table 3).

### 3.4. Association between OA and HTN according to the Menopause Status

The association between OA and HTN in the Mp status is shown in Table 4. In the results of binomial logistic regression, it was found that OA diagnosed by a physician was associated with HTN diagnosis in the total participants aged 40–59 women with and without controlling covariates (OR = 2.442, 95% CI: 1.957–3.046, *p* < 0.001; OR = 1.376, 95% CI: 1.083–1.747, *p*=0.009, respectively).

The OA diagnosis showed a significant association with HTN diagnosed by physicians in which the OR of HTN was 2.939 (95% CI: 1.744–4.954, *p* < 0.001) in PreMp and 1.594 (95% CI: 1.243–2.045, *p* < 0.001) in Mp without controlling covariates. In adjusting for covariates, OA was identified as having a significant association with HTN in Mp participants, which showed OR 1.408 (95% CI: 1.092–1.815, *p*=0.008) in the participants with Mp. However, in PreMp participants, the association between OA and HTN was weakened (OR = 1.651, 95% CI: 0.950–2.869, *p*=0.075).

## 4. Discussion

This study demonstrated that OA diagnosed by a physician was significantly associated with HTN diagnosis in middle-aged women (range: 40–59 years) after adjustment for age, smoking, alcohol, and obesity, although the association was weak in premenopausal women.

Several studies described the association between OA and metabolic syndrome [8, 18–20]. Among the results on metabolic syndrome, Lee et al. reported that HTN patients showed a significantly higher prevalence of knee OA in the Korean population. Hart et al. also found that HTN is one of the metabolic factors associated with knee OA [8, 12]. Puenpatom and Victor found that HTN, abdominal obesity, and hyperglycemia were more prevalent in patients with OA than in those without OA [21]. HTN is possible to be an independent risk factor for knee OA. However, because HTN has been known to be related to many confounding factors, it is still controversial that OA is associated with HTN [9, 14].

These associations have been explained by mechanisms such as inflammation, lipid metabolism, cytokines, and vitamin D receptor. OA and metabolic syndrome were reported to share the same mechanisms of inflammation. Metabolic syndrome has been known as a factor leading to the chronic low-grade inflammatory status in joint tissues. Additionally, as cholesterol accumulated in the cartilage, the efflux function of cartilage could be impaired, hence inducing OA [22, 23]. Some studies suggested several shared risk factors, such as aging, obesity, and chronic inflammation, implicated in the plausible mechanisms of the association between HTN and OA, and there are also several reports that the proinflammatory cytokine interleukin-6 has a vital role in HTN and knee OA [11, 24–28]. Additionally, it was shown that polymorphisms in the vitamin D receptor would be possible to be associated with low bone mineral density, OA, and HTN [29, 30].

OA is a serious threat to women's health. The rates of cartilage loss and progression of cartilage defects at the knee have been increased in women more than in men [2]. Moreover, this study reported the results of distinguishing between menopause because OA appears differently depending on the menopause status. Mahajan and Patni reported that menopause is associated with the onset and progression of OA in women, and hand OA was more common in postmenopausal women, even if an important covariate, the age, was controlled [31, 32]. Magliano reported 41% of perimenopausal women had joint pain and stiffness in comparison with 25% of premenopausal women even though the significance was borderline in controlling age. Menopause is also associated with HTN. Cutler et al. reported that women with menopause could have a high prevalence of HTN regardless of their ethnicity [33], though it has not been conclusive to be an independent risk factor for HTN [34].

It was difficult to find studies on the association between OA and HTN based on the menopause status in middle-aged women. The authors emphasize the importance of timely intervention of a preventive approach for skeletal joint protection. To this end, studies for middle-aged, premenopausal women are needed, but they have not been conducted sufficiently. This study reported that a modest change was already underway in premenopausal women, suggesting the necessity for more active research for the middle-aged population, although their change seems to be just beginning.

The results are similar to the positive relation of metabolic syndrome and radiographic knee OA in the Japanese study, including middle-aged women, conducted by Inoue et al. [14]. They showed a significant association, and those strong results may be due to the precise diagnosis of OA through radiography. Age, smoking, drinking, and fitness habit were adjusted in their logistic regression model, but all of the four covariates were not significant. In our analytical model, age, smoking, and drinking were included according to the precedent of this most similar study [14]. Obesity was also included in the model instead of fitness habit because of the strong positive association with knee OA (OR: 1.563; 95% CI: 1.191–2.051) reported in Lee et al.'s study [8].

OA, HTN, and menopause status, these three conditions have shared risk factors such as age, obesity, and sex [22]. However, a comprehensive study investigating the association among these conditions was scarce. This study is unique in this part. Another strength of our research is the representativeness of the study sample, which is possible to provide credence to the result validity. Also, several confounders were controlled in our analysis to guarantee the robustness of the results.

There are several limitations to interpreting the results of this study. Firstly, it was impossible to demonstrate any causal relationship between OA and HTN in women because this study had a cross-sectional design. Secondly, it was not able to adjust whether the patients with OA and HTN diagnosed by the physician were on treatment or not due to data limitation. There was no consideration that it was surgical menopause or natural menopause, and it was not known whether hormone replacement therapy or any treatment for menopausal symptoms was performed. Also, the location of OA, such as the knee, lumber, or hand, was not clear. Another limitation is the lack of uric acid information. Uric acid has been introduced as an inflammatory marker for cardiovascular diseases and metabolic syndrome. Besides, hyperuricemia can induce gout arthropathy, which might be confused with osteoarthritis [35]. Therefore, information on uric acid would be helpful to clarify the results of this article. However, uric acid could not be used because it was not obtained from that year's data. In the future, it would be needed to perform a more comprehensive study to overcome these limitations.

## 5. Conclusion

To our knowledge, this is the first report that explored the association between OA diagnosis and HTN according to the menopausal status, including the premenopausal group. As a result, middle-aged women with HTN have higher risk for OA than the normotensives, and this association was more apparent among postmenopausal women.

In further study, more clear explanations of these mechanisms are needed, including prospective research, to assess a causal association between OA and HTN to develop preventive strategies for women in middle age to achieve active life in their elderly.

## Figures and Tables

**Table 1:** Characteristics of the groups with and without OA.

Characteristics		Non-OA (*n* = 4,148) *n* (%)	OA (*n* = 479) *n* (%)
Age (years)	40–49	2141 (51.6)	77 (16.1)
	50–59	2007 (48.4)	402 (83.9)

Smoking	Smoking	208 (5.0)	29 (6.1)
	Past	142 (3.4)	26 (5.5)
	Nonsmoking	3781 (91.5)	421 (88.4)

Alcohol consumption	No	538 (13.0)	65 (13.7)
	<1/m^a^	1772 (42.9)	215 (45.1)
	>1/m^a^	1823 (44.1)	196 (41.2)

Menopause	No	2289 (55.2)	104 (21.7)
	Yes	1859 (44.8)	375 (78.3)

Hypertension	No	3604 (86.9)	350 (73.1)
	Yes	544 (13.1)	129 (26.9)

BMI (Kg/m^2^)	Low (<18.5)	138 (3.3)	8 (1.7)
	Normal (18.5 to <25)	2884 (69.6)	242 (50.5)
	Obesity (≥25)	1119 (27.0)	229 (47.8)

Clinical		Mean (SD)	Mean (SD)
	Age (years)	49.2 (5.7)	53.9 (4.3)
	EQ-5D	1.0 (0.1)	0.9 (0.1)
	SBP (mmHg)	114.7 (15.9)	119.3 (17.0)
	DBP (mmHg)	75.2 (9.7)	77.1 (10.1)
	FBS (mg/dL)	97.8 (21.6)	101.0 (27.5)
	TG (mg/dL)	118.7 (91.7)	134.3 (88.4)
	Chol (mg/dL)	197.0 (35.0)	204.3 (38.1)
	HDL-C (mg/dL)	54.6 (12.4)	53.7 (13.4)
	AST (IU/L)	21.0 (10.0)	23.0 (16.1)
	ALT (IU/L)	18.8 (14.7)	21.3 (15.9)
	CREA (mg/dL)	0.7 (0.2)	0.7 (0.1)

SD: standard deviation; OA: osteoarthritis; EQ-5D: euro quality of life-5 dimensions; SBP: systolic blood pressure; DBP: diastolic blood pressure; FBS: fasting blood sugar; TG: triglyceride; Chol: cholesterol; HDL-C: high-density lipoprotein cholesterol; AST: aspartate aminotransferase; ALT: alanine aminotransferase; CREA: creatinine. ^a^1/m = 1 glass per month.

**Table 2:** Characteristics of the premenopausal and menopausal groups according to the osteoarthritis status.

Characteristics		Total *n*	Non-OA (*n* = 4,148)		*p* value	Total *n*	OA (*n* = 479)		*p* value
			PreMp (*n* = 2,289) *n* (%)	Mp (*n* = 1,859) *n* (%)			PreMp (*n* = 104) *n* (%)	Mp (*n* = 375) *n* (%)	
Age (years)	40–49	2141	1943 (84.9)	198 (10.7)	<0.001	77	65 (62.5)	12 (3.2)	<0.001
	50–59	2007	346 (15.1)	1661 (89.3)		402	39 (37.5)	363 (96.8)	

Smoking	Smoking	208	116 (5.1)	92 (5.0)	0.042	29	8 (7.8)	21 (5.6)	0.705
	Past	142	93 (4.1)	49 (2.6)		26	6 (5.8)	20 (5.4)	
	Nonsmoking	3781	2072 (90.8)	1709 (92.4)		421	89 (86.4)	332 (89.0.)	

Alcohol consumption	No	538	232 (10.2)	306 (16.5)	<0.001	65	8 (7.8)	57 (15.3)	0.040
	<1/m^a^	1772	934 (40.9)	838 (45.3)		215	43 (41.7)	172 (46.1)	
	>1/m^a^	1823	1116 (48.9)	707 (38.2)		196	52 (50.5)	144 (38.6)	

HTN	No	3604	2129 (93.0)	1475 (79.3)	<0.001	350	85 (81.7)	265 (70.7)	0.015
	Yes	544	160 (7.0)	384 (20.7)		129	19 (18.3)	110 (29.3)	

BMI (Kg/m^2^)	Low (<18.5)	138	93 (4.1)	45 (2.4)	0.004	8	3 (2.9)	5 (1.3)	0.217
	Normal (18.5 to <25)	2884	1602 (70.0)	1282 (69.1)		242	58 (55.8)	184 (49.1)	
	Obesity (≥25)	1119	592 (25.9)	527 (28.4)		229	43 (41.3)	186 (49.6)	

Clinical			Mean (SD)	Mean (SD)			Mean (SD)	Mean (SD)	
	Age	4148	45.4 (3.9)	54.1 (3.6)	<0.001	479	48.1 (3.9)	55.5 (2.8)	<0.001
	EQ-5D	4146	1.0 (0.1)	1.0 (0.1)	<0.001	479	0.9 (0.1)	0.9 (0.1)	0.482
	SBP (mmHg)	4140	111.9 (14.8)	118.0 (16.6)	<0.001	478	114.8 (14.0)	120.6 (17.6)	0.002
	DBP (mmHg)	4140	74.3 (9.7)	76.2 (9.6)	<0.001	478	75.6 (9.7)	77.5 (10.2)	0.088
	FBS (mg/dL)	3981	96.0 (18.8)	100.0 (24.5)	<0.001	453	97.2 (20.9)	102.1 (29.0)	0.117
	TG (mg/dL)	3982	109.0 (77.0)	130.7 (106.0)	<0.001	453	125.8 (91.0)	136.8 (87.6)	0.274
	Chol (mg/dL)	3982	191.2 (32.5)	204.3 (36.5)	<0.001	453	197.6 (37.1)	206.2 (38.7)	0.046
	HDL-C (mg/dL)	3982	55.4 (12.5)	53.7(12.3)	<0.001	453	55.1 (13.2)	53.4(13.5)	0.250
	AST (IU/L)	3982	19.4 (9.4)	23.0 (10.4)	<0.001	453	20.9 (6.9)	23.6 (17.9)	0.143
	ALT (IU/L)	3982	16.9 (15.0)	21.1 (14.1)	<0.001	453	19.6 (11.6)	21.7 (16.9)	0.226
	CREA (mg/dL)	3976	0.7 (0.1)	0.7 (0.2)	0.185	452	0.7 (0.1)	0.7 (0.1)	0.380

OA: osteoarthritis; Mp: menopause; HTN: hypertension; SD: standard deviation; EQ-5D: euro quality of life-5 dimensions; SBP: systolic blood pressure; DBP: diastolic blood pressure; FBS: fasting blood sugar; TG: triglyceride; Chol: cholesterol; HDL-C: high-density lipoprotein cholesterol; AST: aspartate aminotransferase; ALT: alanine aminotransferase; CREA: creatinine. ^a^1/m = 1 glass per month.

**Table 3:** Characteristics of the groups with and without hypertension according to the menopause status.

Characteristics		Total *n*	PreMp (*n* = 2,408)		*p* value	Total *n*	Mp (*n* = 2,219)		*p* value
			Non-HTN (*n* = 2,228) *n* (%)	HTN (*n* = 180) *n* (%)			Non-HTN (*n* = 1,726) *n* (%)	HTN (*n* = 493) *n* (%)	
Age (years)	40–49	2018	1900 (94.2)	118 (5.8)	<0.001	200	174 (87.0)	26 (13.0)	<0.001
	50–59	390	328 (84.1)	62 (15.9)		2019	1552 (76.9)	467 (23.1)	

Smoking	Smoking	124	112 (90.3)	12 (9.7)	0.613	113	86 (76.1)	27 (23.9)	0.801
	Past	99	92 (92.9)	7 (7.1)		69	52 (75.4)	17 (24.6)	
	Nonsmoking	2176	2017 (92.7)	159 (7.3)		2026	1579 (77.9)	447 (22.1)	

Alcohol consumption	No	378	349 (92.3)	29 (7.7)	0.574	434	335 (77.2)	99 (22.8)	0.097
	<1/m^a^	903	837 (92.7)	66 (7.3)		835	673 (80.6)	162 (19.4)	
	>1/m^a^	877	822 (93.7)	55 (6.3)		580	441 (76.0)	139 (24.0)	

OA	No	2303	2142 (93.0)	161 (7.0)	<0.001	1845	1462 (79.2)	383 (20.8)	<0.001
	Yes	105	86 (81.9)	19 (18.1)		374	264 (70.6)	110 (29.4)	

BMI (Kg/m^2^)	Low (<18.5)	96	95 (99.0)	1 (1.0)	<0.001	50	49 (98.0)	1 (2.0)	<0.001
	Normal (18.5 to <25)	1668	1578 (94.6)	90 (5.4)		1458	1228 (84.2)	230 (15.8)	
	Obesity (≥25)	639	550 (86.1)	89 (13.9)		709	447 (63.0)	262 (37.0)	

Clinical			Mean (SD)	Mean (SD)			Mean (SD)	Mean (SD)	
	Age	2408	45.3 (3.7)	47.8 (3.8)	<0.001	2219	54.1 (3.6)	55.2 (3.1)	<0.001
	EQ-5D	2406	1.0 (0.1)	1.0 (0.1)	0.054	2219	0.9 (0.1)	0.9 (0.1)	0.018
	SBP (mmHg)	2401	111.0 (14.4)	124.3 (14.4)	<0.001	2217	115.7 (15.7)	128.4 (16.6)	<0.001
	DBP (mmHg)	2401	73.7 (9.5)	81.8 (9.9)	<0.001	2217	75.2 (9.3)	81.2 (9.9)	<0.001
	FBS (mg/dL)	2316	95.3 (17.7)	104.6 (28.0)	<0.001	2118	98.6 (21.6)	106.9 (35.0)	<0.001
	TG (mg/dL)	2317	106.7 (74.2)	146.9 (104.4)	<0.001	2118	126.1 (87.7)	152.2 (144.8)	<0.001
	Chol (mg/dL)	2317	191.2 (32.3)	194.1 (37.1)	0.325	2118	207.3 (36.4)	195.4 (37.0)	<0.001
	HDL-C (mg/dL)	2317	55.7 (12.5)	51.5 (11.8)	<0.001	2118	54.4 (12.6)	51.1 (12.0)	<0.001
	AST (IU/L)	2317	19.2 (8.6)	22.7 (15.2)	0.003	2118	22.6 (9.4)	25.0 (18.5)	0.006
	ALT (IU/L)	2317	16.5 (13.8)	23.3 (22.8)	<0.001	2118	20.2 (13.0)	24.7 (18.9)	<0.001
	CREA (mg/dL)	2317	0.7 (0.1)	0.7 (0.2)	0.144	2118	0.7 (0.1)	0.7 (0.4)	<0.001

OA: osteoarthritis; Mp: menopause; HTN: hypertension; SD: standard deviation; EQ-5D: euro quality of life-5 dimensions; SBP: systolic blood pressure; DBP: diastolic blood pressure; FBS: fasting blood sugar; TG: triglyceride; Chol: cholesterol; HDL-C: high-density lipoprotein cholesterol; AST: aspartate aminotransferase; ALT: alanine aminotransferase; CREA: creatinine. ^a^1/m = 1 glass per month.

**Table 4:** Association between hypertension and osteoarthritis among total, premenopause, and menopause groups from logistic regression analysis.

		Unadjusted OR	*p* value	Adjusted^a^ OR	*p* value
Total	Non-HTN	1.000	—	1.000	—
	HTN	2.442 (1.957, 3.046)	<0.001	1.376 (1.083, 1.747)	0.009

PreMp (*n* = 2,408)	Non-HTN	1.000	—	1.000	—
	HTN	2.939 (1.744, 4.954)	<0.001	1.651 (0.950, 2.869)	0.075

Mp (*n* = 2,219)	Non-HTN	1.000	—	1.000	—
	HTN	1.594 (1.243, 2.045)	<0.001	1.408 (1.092, 1.815)	0.008

^a^Adjusted for age, smoking, alcohol consumption, and BMI (continuous variable). OR: odds ratio; Mp: menopause; HTN: hypertension.

## Data Availability

The data used to support the findings of this study are available from the website of the Korea National Health and Nutrition Examination Survey (https://knhanes.cdc.go.kr/knhanes/main.do).
